# Predictors of mortality among HIV exposed infants at University of Gondar Comprehensive Specialized Hospital, Northwest Ethiopia

**DOI:** 10.1186/s13052-019-0740-9

**Published:** 2019-11-07

**Authors:** Chalachew Adugna Wubneh, Aklilu Endalamaw, Nigusie Birhan Tebeje

**Affiliations:** 1 Department of Pediatrics and Child Health Nursing, School of Nursing, College of Medicine and Health Science, University of Gondar, Gondar, Ethiopia; 2 Department of Pediatrics and Child Health Nursing, College of Medicine and Health Science, Bahir Dar University, Bahir Dar, Ethiopia; 3 Unit of Community Health Nursing, School of Nursing, College of Medicine and Health Science, University of Gondar, P.O.BOX=196, Gondar, Ethiopia

**Keywords:** HIV exposed infant, Mortality, Predictors, Ethiopia

## Abstract

**Background:**

In the era of highly active antiretroviral therapy, vertical HIV transmission has been decreased. This may increase fertility desire of HIV infected women and an increasing number of HIV exposed infants as a result. A high probability of mortality among HIV exposed infants was reported across different countries. However, few studies are found on mortality of HIV exposed infants, in particular, no study was conducted before in the current study area.

**Methods:**

Institution based retrospective cohort study from July 2013 to December 2017 was conducted. A total of 408 HIV exposed children were selected through simple random sampling technique. Data were extracted from registration book by using data extraction tool, which is adapted from the Ethiopian Federal Ministry of Health HIV exposed infant follow-up form. Kaplan–Meier survival curve was used to show the probability of mortality rate. Bivariable and multivariable cox regression models were used to identify predictors of mortality.

**Results:**

Overall mortality rate was found to be 8.88 (95% CI: 6.36–12.36) per 100 child-year. Infant with death of at least one parent (AHR = 3.32; 95% CI: 1.503–7.32), non-exclusive breastfeeding (AHR = 0.10; 95% CI: 0.037–0.302), growth failure (AHR = 2.9; 95% CI: 1.09–8.09), presence of sign and symptom of HIV infection (AHR = 2.99; 95% CI: 1.33–6.74), and low birth weight (AHR = 2.6; 95% CI: 1.007–6.78) were found to be predictors of infant mortality.

**Conclusions:**

Mortality of HIV exposed infants was high in Ethiopia. Prevention of the occurrence of HIV infection symptom, growth failure, and low birth weight is essential and further treat early whenever they occurred. Still, behavioral change interventions on mother who practice non-exclusive breastfeeding are indicated. Especial care for orphan infants is required due to their nature of vulnerability to varieties of health problem.

## Background

Based on the Joint United Nations Program on HIV/AIDS (UNAIDS) report in 2018, 36.9 million people living with Human Immunodeficiency Virus (HIV). Of which, more than 50% were women and 90% of HIV positive pregnant women live in sub-Saharan Africa [1]. According to the global plan towards the elimination of new HIV infections among children mother to child HIV transmission has been reduced by 60% and the number of ADIS related child death was 49,000 as a referee of 2015 report [2]. It shows child mortality continued to be a persistent global problem. In 2015, an estimated 1.2 million babies were born from HIV-positive mothers [3, 4]. Those children born from HIV positive mothers are at higher risk of death [5]. Infant health is one of the indicators of countries developmental progress.

Consequently, at the international and national level, different strategies have been implemented to reduce mortality of children born from HIV positive mother. Notably, the World Health Organization (WHO) four prongs Prevention of Mother-to-Child Transmission (PMTCT) of HIV approach considered a key entry point of care for HIV positive pregnant, laboring and lactating women and their children is to be an infant mortality preventive strategy. These include primary prevention of HIV infection, prevention of unintended pregnancy among HIV positive women, prevention of HIV transmission from infected women to their children and treatment, care and support of HIV infected women, their newborn and families [6, 7]. Moreover, the Ethiopian ministry of health initiated ‘option B+’ PMTCT approach since 2013 [6]. This approach recommends initiating Antiretroviral Therapy (ART) for all pregnant, laboring and lactating women, improving ART coverage, test to HIV and start HAART for all HIV positive children, early diagnosis for HIV exposed infant and improving health service utilization [6, 8].

Despite these much of interventions, the mortality of children who born from HIV positive mother is the public health problem for African and other less developed countries [9]. The incidence of child mortality reported 5/1000 person-year in India [10] and 150 per 1000 person-years in Zimbabwe [11]. It is also reported in Uganda (3.9%) [12], Cameron (23.9%) [13], Malawi (6.3–9.3%) [14], Rwanda (4.8%) [15], and Kenya 39% [16].

The High mortality of infants born from HIV positive mother has been attributed to different predictors. Some of the predictors identified in the previous studies from different countries include advanced maternal disease [17], HIV infection of the child [11, 14, 18], low birth weight [14], maternal survival status [19], early and abrupt breastfeeding cessation and mixed feeding [13, 15, 20], and prematurity [13].

In Ethiopia, there is no study done to estimate the mortality of HIV exposed infants before. Therefore, we assessed mortality and its predictors among HIV exposed infants at the University of Gondar comprehensive specialized hospital, Northwest Ethiopia.

## Method

### Study design and period

A retrospective cohort study from July 2013 to December 2017 at the University of Gondar Comprehensive Specialized Hospital was conducted.

### Study setting

University of Gondar Comprehensive Specialized Hospital is a teaching hospital, which serves more than five million people in the catchment area. This hospital has started to provide ART for children, adult and PMTCT service for women and their child since 2005. The hospital has started a new PMTC service since 2013 which is ‘option B+’ that recommends treating all HIV positive pregnant, laboring and lactating women and starting follow-up for newborn up to 18 months.

### Source and study population

All infants less than 18 months of age who were born from HIV positive mother.

#### Inclusion

Infants born from HIV positive mother and have a follow-up on the study area were included.

#### Exclusion criteria

Infants and their mothers with incomplete baseline data were excluded.

### Sampling technique and procedures

Those HIV exposed infants on follow-up have successive registration. Therefore, the number of samples was selected using the computer generated number from the database. Based on the selected child card number, child and mother-pair chart was accessed and retrieved. There were 768 HIV exposed infants’ who have a follow-up from July 2013 to December 2017. By using simple random sampling technique, 423 mother-child cards were retrieved.

### Study variable

#### Children variable

**S**ex, birth weight, gestational age at birth, growth pattern, ARV prophylaxis, adherence to cotrimoxazole preventive therapy (CPT), the presence of suggestive sign and symptom of HIV and infant feeding type.

#### Parent variable

Age, marital status, educational status, residence, number of birth for the mother and father HIV status, ANC follow up, ART staring time, baseline CD4 count, Baseline WHO clinical stage, place of delivery, mode of delivery and parent survival status.

### Operational definitions

#### HIV exposed infants

Children born from HIV positive mother.

#### Lost to follow-up

Infants missing their appointment for more than three months.

#### Suggestive sign and symptom of HIV infection

The presence of at least one abnormal finding suggestive of HIV infection, which includes purulent ear discharge, pneumonia/ lower respiratory infection, persistence diarrhea, and persistence fever.

### Data collection tool and procedures

Data were extracted by using data extraction tool customized from Ethiopian federal ministry of health HIV exposed infant follow-up form that is prepared in the English language. Data were collected from HIV exposed infant and mother’s chart and registration book. Three BSc nurse who has PMTCT training collected the required data.

### Data quality control measures

Data abstraction tool was evaluated and commented by other researchers. For its completeness, the data extraction tool was check from chart and follow-up registration book. A pretest was conducted on 10% of the sample size at the University of Gondar Comprehensive Specialized Hospital. Based on the pretest some modification on the data collection tool was made trained BSc nurses were recruited. One-day training was given to data collectors. The data collection process was closely monitored by one MSc nurse supervisor.

### Data processing and analysis

Data were checked and coded and entered into EPI info version 7.2.2.6. Then, data were exported to SPSS version 20. Descriptive statistics were used to analyze the socio-demographic characteristics and proportion of mortality. Kaplan–Meier survival curve was used to see the probability of child mortality. Bivariable and multivariable Cox regression models were used to identify predictors of mortality. Those variables having *P*-value ≤0.2 in the Bivariable analysis were entered into the multivariable model. The Cox proportional hazard model assumption was checked by using Schoenfeld residuals test. The adjusted hazard ratio (AHR) with a 95% confidence interval (CI) was calculated. *P*-value ≤0.05 was considered as statistically significant.

### Follow up and outcome ascertainment

Starting from 2013 the University of Gondar compressive specialized hospital have HIV exposed child and mother cohort up to the age of 18 months. We use this cohort data retrospectively for this study. Study participants have different follow-up period and different entrance time. The mortality was reported as the death of the child starting from follow-up to the end of 18 months and as register from the follow-up form. It is difficult to relate the exact cause of death because of the quality of documentation. It is not cause specific mortality rate but death related to accident or trauma is not reported on the format.

### Ethical considerations

Ethical clearance was obtained from the University of Gondar institutional ethical review committee. To maintain the confidentiality of the study participants’ information and identifiers like name and medical record number was removed from the data extraction tool. Confidentiality of the information was maintained throughout the research process.

## Result

### Socio-demographic characteristics of the parent

A total of 408 mother-child- pairs’ card have included in the study which provided a 96.4% response rate. Most of (81.9%) of mothers were from urban. Majority (72.5%) of mothers were married. Regarding religion, 90% were Orthodox Christian. More than three-fourths of mothers (77%) attended primary school level and above (Table 1).

**Table 1:** Socio-demographic characteristics of parent at University of Gondar comprehensive specialized hospital, from July 2013–December 2017

Characteristic of the parent	Number	Percent (%)
Age of mother		
Less than 20 years	6	1.5
20–25 year	65	15.9
26–30 year	180	44.1
31 year and above	157	38.5
Residence of the mother		
Urban	334	81.9
Rural	74	18.1
Marital status of the mother		
Married	296	72.5
Unmarried	112	27.5
Occupation of the mother		
Government employed	111	27.2
Non-employed	297	72.8
Religious of the mother		
Orthodox Christian	367	90
Muslim	38	9.3
Protestant	3	0.7
Educational level of the mother		
Unable to read and write	94	23
Primary education	136	33.3
Secondary education	114	27.9
College and above	64	15.7
Number of birth		
Only one	84	20.6
2–4	302	74
Five and above	22	5.4
Survival status of the parent		
Both parent alive	378	92.6
Parent dead	30	7.4
HIV status of the father		
Positive	285	69.9
Negative	69	16.9
Unknown	54	13.2

### Socio-demographic and health-related characteristics of HIV exposed infants

From the total 408 children, more than half (52%) were male. The mean birth weight of the infants was 2.8 kg. Majority (90.7%) of the infants had term gestational age at birth. Majority of infants (93.9%) were enrolled to PMTCT clinic with the age of 45 days. All of the infants had started ART prophylaxis (Nevirapine (NVP) according to the schedule and (99%) of them had good adherence to cotrimoxazole preventive therapy (Table 2).

**Table 2:** Socio-demographic and health related characteristics of HIV exposed infants at University of Gondar compressive specialized hospital from July 2013–December 2107

Characteristic	Number	Percent (%)
Sex of the child		
Male	212	52
Female	196	48
Birth weight		
< 2.5 Kg < < 2.5 Kg	68	16.7
≥ 2.5 Kg	340	83.3
Gestational age at birth		
Term	370	90.7
Preterm	38	9.3
Growth pattern		
Normal	381	93.4
Growth failure	27	6.6
Feeding practice in first 6 months		
EBF	390	95.6
Non-EBF	18	4.4
Symptom and sign suggestive of HIV		
Yes	29	7.1
No	379	92.9

### Pregnancy, delivery, and clinical characteristics of HIV positive mothers

Most of the mothers (94.9%) had attended at least one antenatal care visit for the current pregnancy. Among mothers attended ANC, (90.7%) were screened for sexually transmitted infections. Nearly 4 % (3.8%) of mothers screened for sexually transmitted infections were reactive. The large proportion of mothers (82.3%) gave birth through spontaneous vaginal delivery without episiotomy (Table 3).

**Table 3:** Pregnancy, delivery and other clinical characteristic of HIV positive mothers at University of Gondar compressive specialized hospital from July 2013–December 2017

Characteristics	Number	Percent (%)
Antenatal care		
Attended ANC	387	94.9
No ANC	21	5.1
Number ANC visit		
1 and 2	68	17.8
3 and 4	242	62.7
5 and above	75	19.5
STI screening		
Non-reactive	370	90.7
Reactive	15	3.7
Not done	23	5.6
Place of delivery		
Hospital	364	89.2
Health center	31	7.6
Home	13	3.2
Mode of delivery		
SVD	336	82.3
SVD with episiotomy	15	3.7
CS	57	14
ART starting time		
Before this pregnancy	234	57.4
During this pregnancy	163	40
After delivery	11	3
CD4 cell when ART start		
CD4 cell ≤350	178	43.6
CD4 cell > 350	230	56.4
WHO stage when ART start		
stage I &II	370	90.7
Stage III & IV	38	9.3

### Infant mortality

**Fig. 1:**
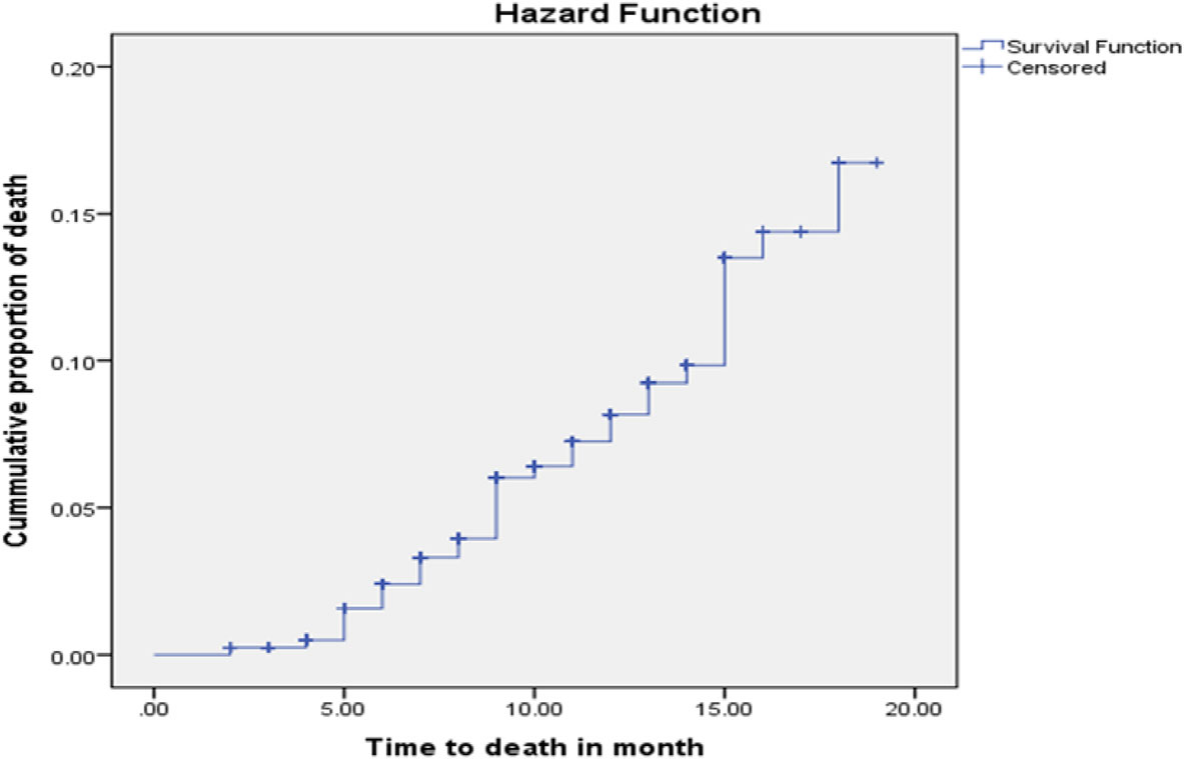
Kaplan-Meier curve of mortality proportion for HIV exposed infants at University of Gondar Comprehensive Specialized Hospital, Northwest Ethiopia, and July 2013–December 2017

Among 408 children followed for a median of 12 months, 35(8.6%) died. The overall mortality rate was 8.88 (95% CI: 6.36–12.36) per 100 child-year. Among the overall death, more than half were male (60%). Nearly half (45.7%) of the death occurred between the age of 6 to 12 month (Fig. 1) (Table 4).

**Table 4:** Bivariable and multivariable cox regression analysis of predictors of mortality among HIV exposed infants at University of Gondar Comprehensive Specialized Hospital, Northwest Ethiopia, July 2013 December 2017

Characteristics	Death	Censored	CHR (95% CI)	AHR (95% CI) P
Marital status of mother				
Married	18	278	1	1
Unmarried	17	95	2.43 (1.25–4.711)	0.96 (0.45–2.06) 0.9
Gestational age at birth				
Term	22	348	1	1
Preterm	13	25	5.93 (2.98–11.77)	1.65 (0.64–4.22) 0.29
Feeding practice in the first 6 month				
EBF	29	361	1	1
Non-EBF	6	12	9.4 (3.85–22.96)	0.10 (0.037–0.302) < 0.0001
Parent survival status				
Both alive	23	355	1	1
At least one of dead	12	18	7.28 (3.6214.63)	3.32 (1.5–7.32) 0.003
Growth pattern				
Normal	29	352	1	1
Growth failure	6	21	3.95 (1.64–9.54)	2.97 (1.09–8.09) 0.03
Symptom and sign suggestive of HIV				
Yes	13	16	8.034 (4.042–15.98	2.99 (1.33–6.71) 0.008
No	22	357	1	1
Birth weight				
< 2.5 Kg	17	51	0.203 (0.105–0.394)	2.6 (1.007–6.78) 0.04
≥ 2.5 Kg	18	322	1	1
CD4 cell when ART start				
CD4 cell ≤350	22	156	2.24 (1.13–4.44)	0.049 (0.226–1.064) 0.07
CD4 cell > 350	13	217	1	1

### Predictors of mortality

In the bivariable Cox regression analysis, gestational age at birth, growth pattern, marital status of the mother, maternal CD4 cell count at ART initiation, parent survival status, presence of suggestive sign and symptom of HIV infection, infant feeding practice in the first 6-month, and birth weight were associated with infant mortality. However, in multivariable Cox regression analysis, birth weight, presence of suggestive sign and symptom of HIV infection, infant feeding practice in the first 6-month, growth pattern, and parent survival status were statistically significant predictors of child mortality. Based on Cox regression analysis, infants whose weight less than 2.5 Kg at birth was 2.6 (95% CI: 1.007–6.78) times at high risk of death compared with infants who had a birth weight of 2.5 Kg and above. Infants who had growth failure were 2.9 (95% CI: 1.09–8.09) times risk for death. Besides, those infants who had a suggestive sign and symptom of HIV infection were 2.9 (95% CI: 1.33–6.74) fold risk for death compared with their counterparts.

**Fig. 2:**
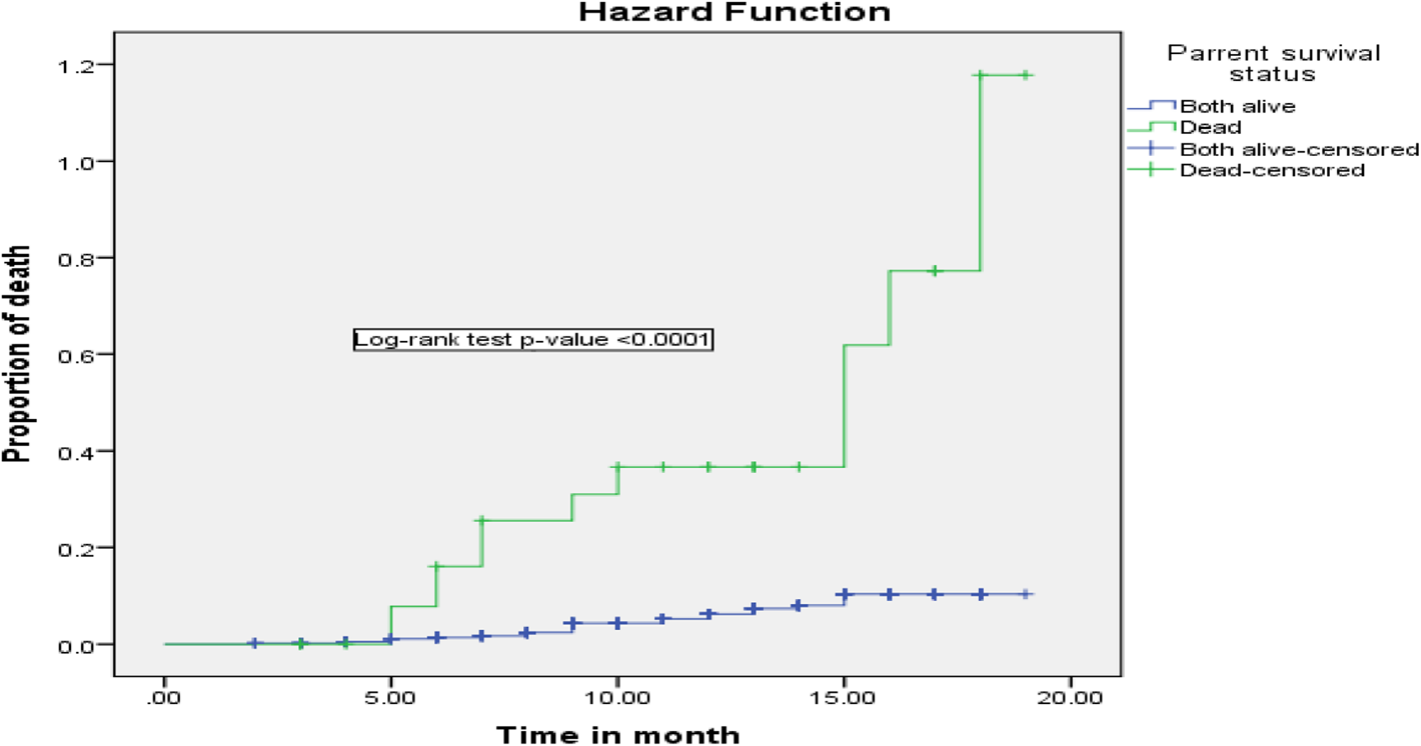
Kaplan-Meier curve of mortality estimate for parent survival status among HIV exposed infants at University of Gondar Comprehensive Specialized Hospital, Northwest Ethiopia, July 2013–December 2017

**Fig. 3:**
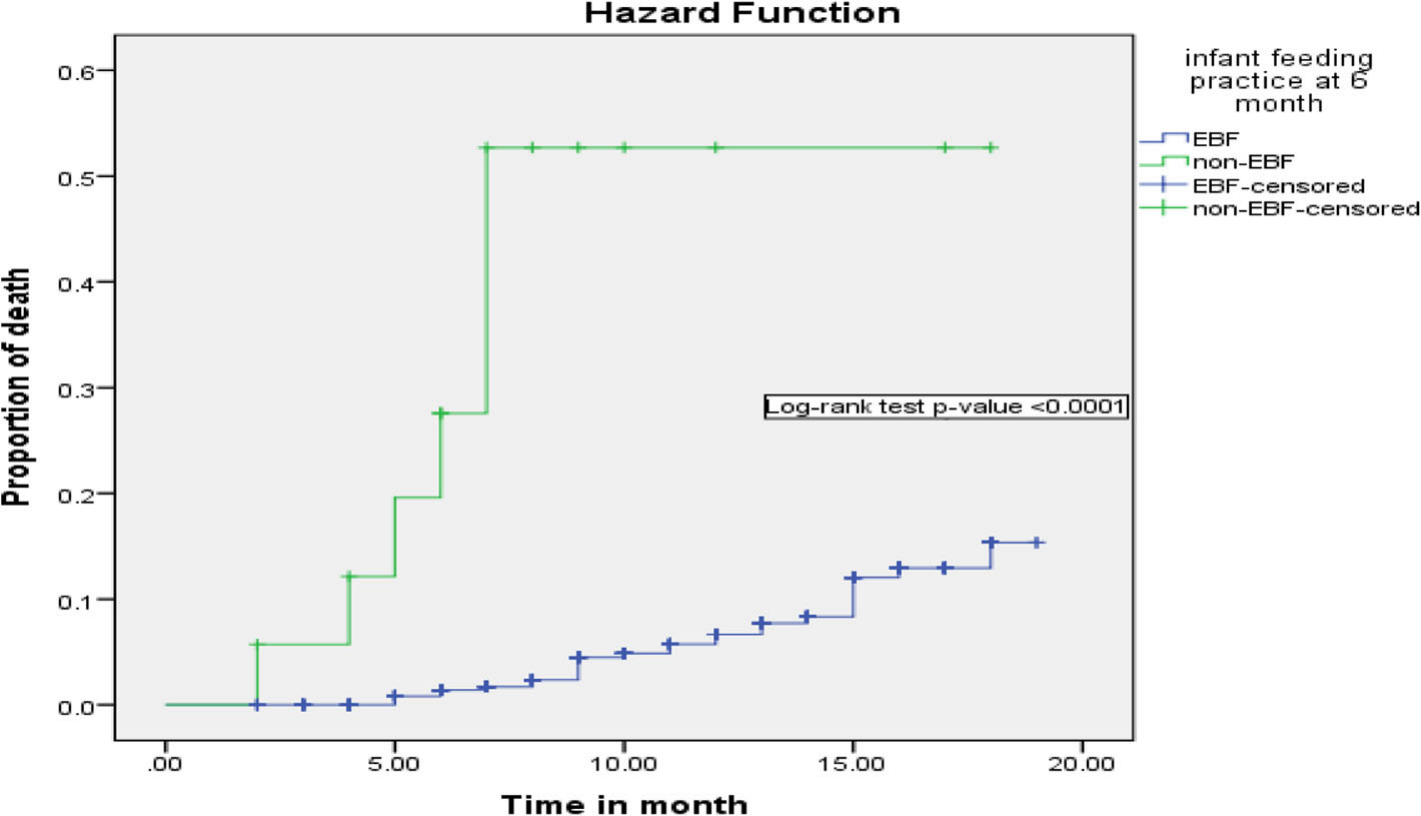
Kaplan-Meier curve of mortality estimate for infant feeding practice among HIV exposed infants at University of Gondar Comprehensive Specialized Hospital, Northwest Ethiopia, July 2013–December 2017

**Fig. 4:**
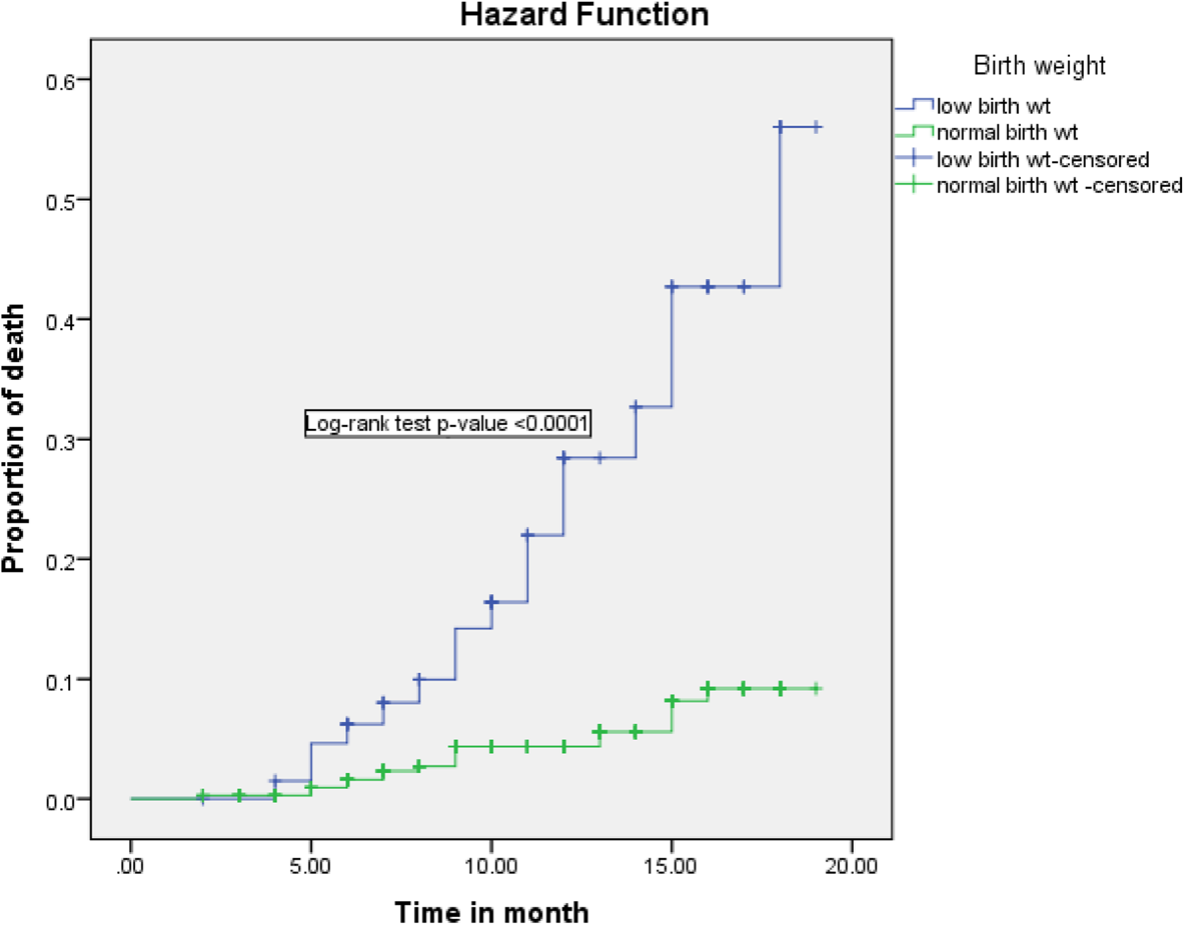
Kaplan-Meier curve of mortality estimate for birth weight among HIV exposed infants at University of Gondar Comprehensive Specialized Hospital, Northwest Ethiopia, July 2013–December 2017

**Fig. 5:**
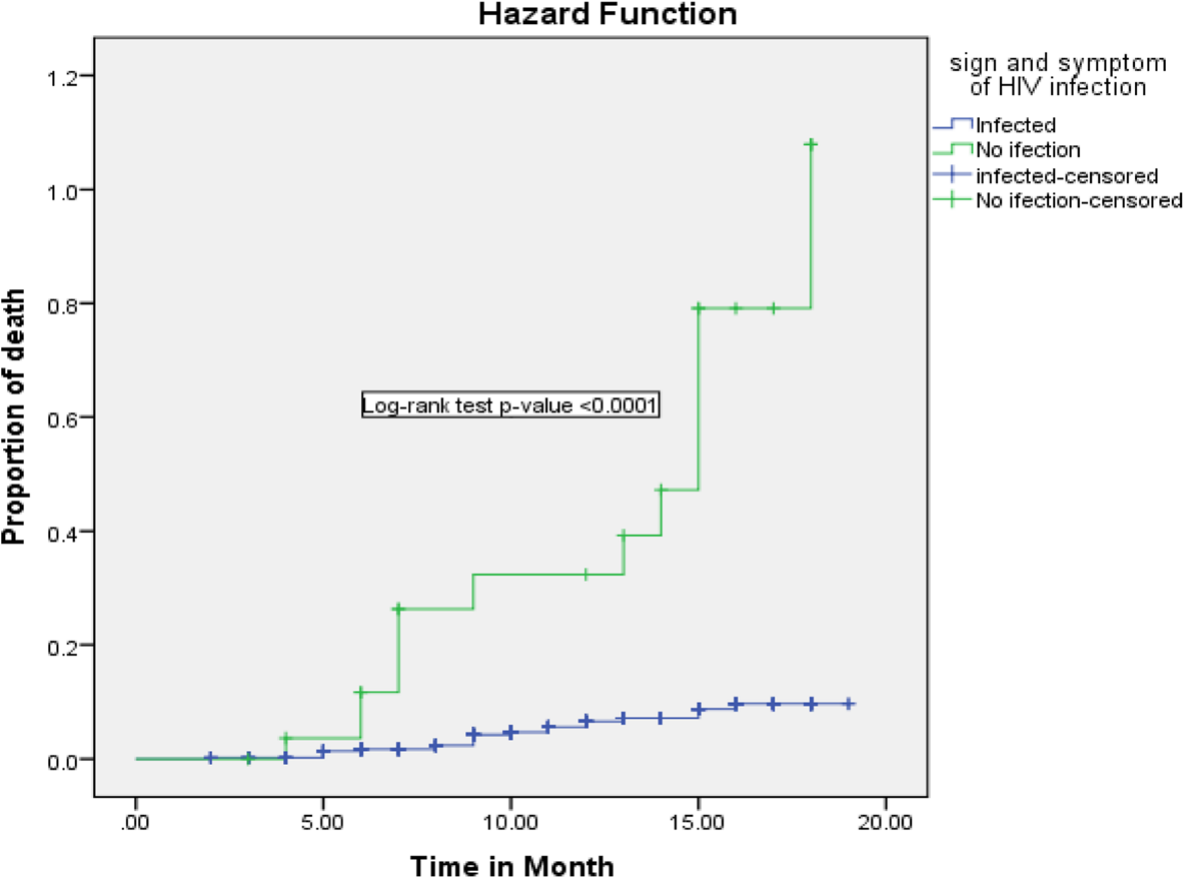
Kaplan-Meier curve of mortality estimate for suggestive sign and symptom of HIV infection among HIV exposed infants at University of Gondar Comprehensive Specialized Hospital, Northwest Ethiopia, July 2013–December 2017

**Fig. 6:**
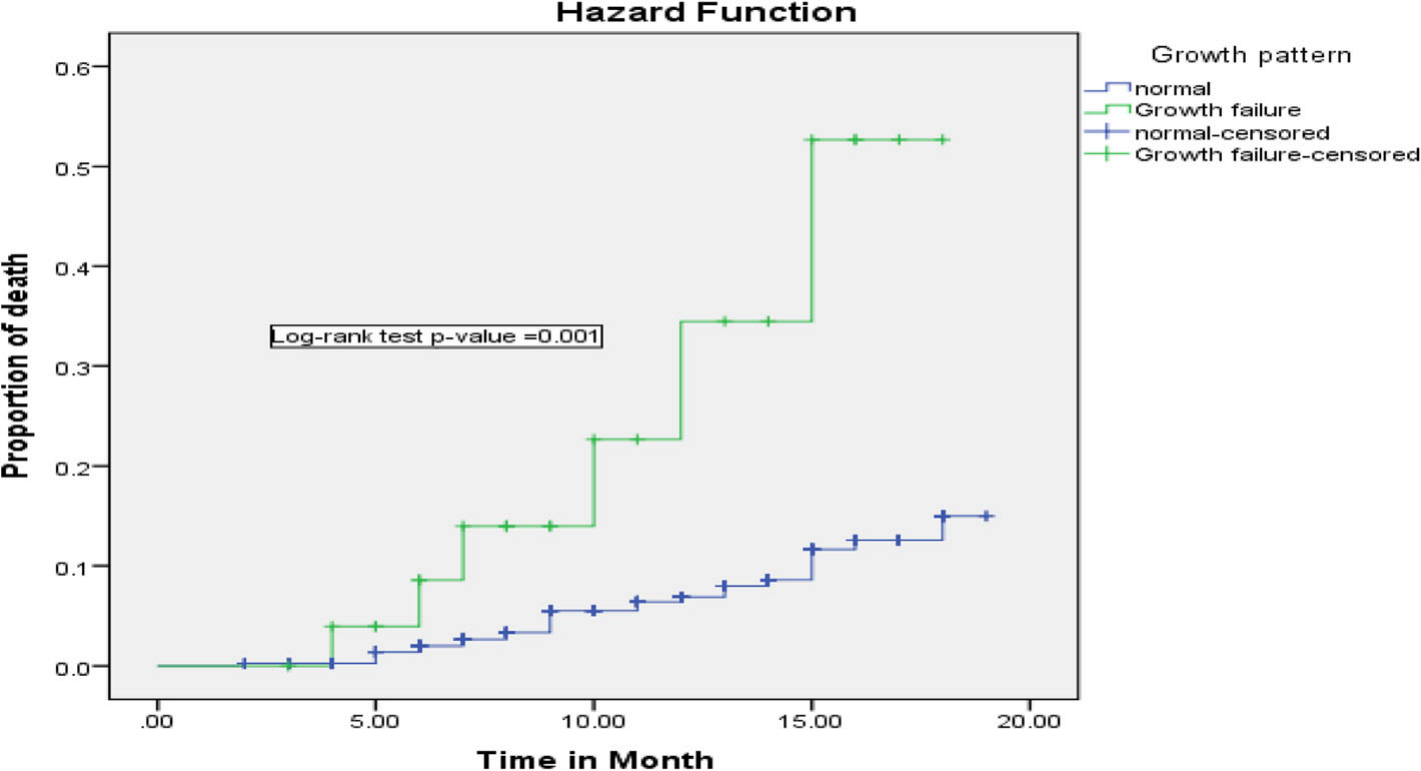
Kaplan-Meier curve of mortality estimate for growth pattern among HIV exposed infants at University of Gondar Comprehensive Specialized Hospital, Northwest Ethiopia, July 2013–December 2017

Regarding infant feeding practice in the first 6-month of life, being exclusive breastfeeding can reduce infant death by 90% (AHR = 0.10; 95% CI: 0.037–0.302) as compared to those infants on non-exclusive breastfeeding. Besides, those infants lost at least one of their parents by death was 3.3 (95% CI: 1.503–7.32) times high risk of death when compared with infants whose parent alive (Figs. 2, 3, 4, 5 and 6).

## Discussion

This study showed that the overall infants’ mortality rate was 8.88 (95% CI: 6.36–12.36) per 100 child-year. This result is higher from the study conducted in Pune India (2.4/100-person-year) [10], Malawi (3.4/100 per person-year) [14], and Zambia (3.5/100 person-years) infants [20]. The exact reason for this difference in mortality is unknown, but it is probably multifactorial. This variation might be due to different follow-up period implemented. The study in India took 12 months’ follow-up period and 70 days a study in Zambia. The difference in the study population is another source; in Malawi only infants up to 48 weeks of age were included. Other possible explanation might be a high burden of infection [21], childhood illness [22], low coverage of prophylaxis [23, 24], high prevalence of under-nutrition [25, 26], low coverage of immunization [27], mixed feeding practice, pre-lacteal feeding [28], low infrastructure [29], health care facilities related infection [30], poor-bottle feeding hygiene recorded in Ethiopia, particularly in the current study region ( [31]. The expansion of ART initiation during delivery remains a national health priority. Hence, asymptomatic infections are remaining undiagnosed and not managed that increased probability of death.

In the Cox multivariable regression analysis parent death was a 3-fold risk for their child death among HIV exposed infants compared with those infants’ with parent alive. The evidence was supported by other studies from Zimbabwe [17] and a systemic review of HIV exposed uninfected African infant [9]. In Ethiopia, Orphan problems are universal. These children are subjected to abuse, neglect exploitation, and carelessness to bring to health institution early and/or to provide medication as ordered [32]. This is because those infants who lost their mother, father or both would lack quality of care and support, which might expose the child to the nutritional insecurity that leads to growth and development failure and undernutrition. Undernourished children could be vulnerable to infectious disease [33] and it increases the death of infants.

This study showed that infants who were on non-exclusive breastfeeding had at higher risk of death compared with infants on exclusive breastfeeding. This finding supported by studies from Cameron [13], a systemic review of HIV exposed infant studies [31], Zambia [20] and Uganda [12]. Mixed feeding, short duration of breastfeeding, and cessation of breastfeeding predisposes infants to dietary pathogens, diarrhea, and declined immunity [11]. As a result, infant mortality can be occurred more likely.

Infants with low birth weight were at high risk for death compared with infants who have normal birth weight. Finding in Malawi [14] also showed that low birth weight increases child mortality. In Ethiopia, the burden of low birth weight is one of the main problems, 17.3% newborn are below normal birth weight [34]. It exposed the infant to hypoglycemia, hypothermia, mental retardation, and growth failure that exposed to infection and related complication, which further contribute to child mortality.

In this study presence of the suggestive sign and symptom of HIV, infection was one of the predictors of infant mortality. The result was supported by a systemic review on HIV-exposed uninfected African child [9] and a study in Botswana [35]. This may be due to HIV exposed infants are expected to impaired immunity system than HIV unexposed infants. This sign and symptoms are the indications of underdiagnosed and non-managed systemic infections. As infection rate increase the risk of death is high.

Growth failure was one of the predictors of infant mortality which increases the death rate of infants by 2.9 times compared with infants who had normal growth. This evidence was supported by a systemic review conducted on HIV exposed uninfected infant [25]. This may be due to the reason that those infant with growth failure can expose to infection, immunological failure, and increased hospitalization that could increase mortality of infants.

### Limitation of the study

Most of the limitations of the study related with poor documentation system. This study did not show the exact cause of death because of poor recorded patient data; in the chart, it was difficult to access the cause of death for the infants. The study conducted based on registered data; we could not include some variables like immunization status, family income and hospitalization rate in the analysis. The other limitation of this study, it did not have a comparison group from HIV unexposed infants to see the clear contribution of HIV exposure on infant mortality.

## Conclusions and recommendation

Mortality of HIV exposed infants was high in Ethiopia. Prevention of the occurrence of HIV infection symptom, growth failure, and low birth weight is essential and further treat early whenever they occurred. Still, behavioral change interventions on mother who practice non-exclusive breastfeeding are indicated. Especial care for orphan infants is required due to their nature of vulnerability to varieties of health problem. It would be good to fill all the data properly on the chart, registration book and follow-up forms, like immunization status, income, and hospitalization rate of the child.

## Data Availability

All data generated or analyzed during this study are included in this article.

## Abbreviations

AHR: Adjusted Hazard Ratio
AIDS: Acquired Immune Deficiency Syndrome
ANC: Antenatal Care
ART: Antiretroviral Therapy
CD4: Cluster of Differentiation 4
CHR: Crude Hazard Ratio
CI: Confidence Interval
DNA: Deoxyribonucleic Acid
EBF: Exclusive Breast Feeding
HIV: Human Immunodeficiency Virus
PCR: Polymerase Chain Reaction
PMTCT: Prevention of Mother-to-Child Transmission
STI: Sexual Transmitted Infection
UN IGME: United Nations Inter-Agency Group for Child Mortality Estimation
UNAIDS: Joint United Nations Program on HIV/AIDS
WHO: World Health Organization
